# Development of Film-Forming Gel Formulations Containing Royal Jelly and Honey Aromatic Water for Cosmetic Applications

**DOI:** 10.3390/gels9100816

**Published:** 2023-10-13

**Authors:** Sirawut Thewanjutiwong, Patcharin Phokasem, Terd Disayathanoowat, Saranya Juntrapirom, Watchara Kanjanakawinkul, Wantida Chaiyana

**Affiliations:** 1 Department of Pharmaceutical Sciences, Faculty of Pharmacy, Chiang Mai University, Chiang Mai 50200, Thailand; sirawut_t@cmu.ac.th; 2 Office of Research Administration, Chiang Mai University, Chiang Mai 50200, Thailand; patcharin.ph@cmu.ac.th; 3 Department of Biology, Faculty of Science, Chiang Mai University, Chiang Mai 50200, Thailand; terd.dis@cmu.ac.th; 4 Research Center of Deep Technology in Beekeeping and Bee Products for Sustainable Development Goals: SMART BEE SDGs, Chiang Mai University, Chiang Mai 50200, Thailand; 5 Chulabhorn Royal Pharmaceutical Manufacturing Facilities by Chulabhorn Royal Academy, Phlu Ta Luang, Sattahip, Chon Buri 20180, Thailand; saranya.jun@cra.ac.th (S.J.); watchara.kan@cra.ac.th (W.K.)

**Keywords:** film-forming gel, polymers, royal jelly, honey, aromatic water, cosmetic, sterilization

## Abstract

This study aimed to develop a film-forming gel containing honey aromatic water (HW) and royal jelly (RJ) for cosmetic applications as a facial peel-off mask. HW, which is industrial waste from the water-reduction process of honey, was sterilized by autoclaving and filtration through a 0.22 µm membrane. The film-forming gels were developed using various types of film-forming polymers, including polyvinyl alcohol (PVA 117), carboxymethyl cellulose (CMC), and hydroxyethyl cellulose (HEC). The gel formulations were characterized in terms of their external appearance, viscosity, pH, and drying time, whereas the films generated were characterized by a texture analyzer, microscopic investigation, Fourier transform infrared, and an X-ray diffractometer. The findings highlighted that HW has short storage shelf life due to microbial contamination. Sterilizations were required before further product development. The film-forming gel was created by using the combination of PVA 117, CMC, and HEC. HW and RJ were successfully incorporated into the film-forming gel. However, HW resulted in a decrease in the gel viscosity and mechanical properties of its film. Interestingly, the drying time was dramatically decreased, which would be more desirable for its use as a peel-off mask. Furthermore, incorporation of royal jelly enhanced the viscosity of the gels as well as improved the mechanical properties of the film. No effect on the chemical and crystal structure of the films was detected after the incorporation. Therefore, the film-forming gels containing HW and RJ, possessing aesthetic attributes that extended to both the gels themselves and the resultant films, were suitable for use as a peel-off mask.

## 1. Introduction

The film-forming system offers an innovative approach for delivering bioactive compounds to the skin, facilitating applications through both topical and transdermal routes [1]. These systems are uncomplicated, offering benefits such as transparency, non-greasy texture, reduced skin irritation, resistance to wiping off, prolonged adhesion, enhanced dosage adaptability, improved patient adherence, and an appealing aesthetic aspect [2]. Film-forming formulations, upon contacting the skin and leaving a thin transparent film of excipients and drug upon solvent evaporation, resulting in their capability to facilitate controlled and extended drug release onto the skin, can be developed in various forms, encompassing solutions, gels, or emulsions [3,4]. Gels possess unique attributes that grant them advantages over solutions and emulsions. Superior to solutions, the non-drip applications of gels reduce wastage and improves adherence and permeation, resulting in enhanced therapeutic outcomes. Superior to emulsions, gels do not require emulsifiers, potentially mitigating irritations. These qualities make gels optimal for applications in both the cosmeceutical and pharmaceutical sectors.

Honeybees are important livestock that generate revenue for society. Therefore, beekeeping plays a significant role in agricultural and rural development in many countries [2,5,6]. Honey, a product of bees abundant in various phenolic compounds, proteins, and sugars, boasts a spectrum of beneficial health impacts, rendering it a reliable and resilient nutritional source during periods of scarcity [7]. In addition to honey, a diverse array of bee products exists, encompassing beeswax, bee venom, propolis, pollen, and royal jelly [7,8,9]. Honey, a naturally occurring substance that is a supersaturated carbohydrate solution with a variety of qualities, is the most extensively produced and commercially accessible bee product [10,11,12]. China leads as a significant global exporter of bee products, including 124,494 metric tons valued at USD 294 million in total exports for 2019, with approximately 120,800 tons constituting honey and 345 tons being royal jelly [13].

Honey is synthesized by bees through the processes of gathering, preserving, and enzymatic transformation of floral nectar [14,15], with its inherent characteristics, influenced by variables such as moisture content and flavor, intrinsically linked to its compositions [13]. Food quality regulations encompass moisture content, and official standards for honey stipulate a maximum water content of 20% *w*/*w* [16]. Since honey is hygroscopic, honey produced in tropical countries contains higher moisture content than the acceptance criteria. High-moisture honey is prone to fermentation and crystallization, so its moisture content must be reduced to improve its quality and meet the required standards. Water from this water reduction process is a by-product that may become waste without further use. Fascinatingly, this water exhibits a distinctive aroma and flavor profile. On the other hand, honey aromatic water has a mild acidity, which might be due to the presence of bioactive components, possibly organic acids. Consequently, it lends itself well to being utilized as an ingredient in cosmetic products.

Royal jelly, on the other hand, is another bee product that is rich in a variety of nutrients and high in skin-beneficial biological active components. However, the restriction on using royal jelly is its short half-life and the fact that it cannot be stored at ambient temperature for a long time. Normally, royal jelly requires refrigeration or freezing to maintain its quality. Freeze-drying the royal jelly and keeping it in the form of dry powder is another way to prolong its storage. To incorporate royal jelly as the primary active ingredient in a cosmetic product, a film-forming system would be a compelling option. A film-forming system is a product that loses volatile components after application to the skin and forms a film. Apart from being beneficial for preserving the active compounds [17], the residual film also seals the skin, increases skin hydration, and is beneficial for certain skin conditions by improving the delivery through the skin layer [17].

Therefore, the present study aims to develop a film-forming gel containing honey aromatic water and royal jelly for cosmetic applications such as peel-off masks. As honey aromatic water is an industrial by-product that has not been utilized, this water is currently discarded as waste. The present study is the first attempt to use honey aromatic water for cosmetic purposes, therefore redefining the area.

## 2. Results and Discussion

### 2.1. Honey Aromatic Water

Honey aromatic water is a transparent liquid with no color but a slight honey odor. The pH of honey aromatic water is around 4.0. Its acidity is likely due to the organic acid components found in honey, such as acetic, gluconic, and lactic acids, which have been reported as predominant aliphatic organic acids in honey [18]. Among these, gluconic acid has been reported as the predominant organic acid found in all types of honey [19,20]. However, as honey aromatic water is a by-product derived from the distillation of honey, its exact composition can differ based on the source of the honey, processing methods, and various other factors. Aside from predominant aliphatic organic acids, many others have also been found, such as acetic, citric, formic, fumaric, D-glucuronic, glutaric, L-malic, oxalic, propionic, D-quinic, L-tartaric, succinic, etc. [21,22,23].

### 2.2. Sterilization Process of Honey Aromatic Water

After the sterilization processes, honey aromatic water from both the filtration and autoclave processes was the same as non-sterile honey water in terms of external appearance and pH, as shown in Figure S1. However, the sedimentation was detected in non-sterile honey aromatic water and sterile honey aromatic water from autoclaves, whereas the filtration honey aromatic water sample had no sediment. The likely explanation for the observed sediment in the honey aromatic water is the presence of microorganisms or yeast, which can grow, aggregate, and form clusters, biofilms, or sediment [24]. Furthermore, the turbidity or sediment could be attributed to cellular debris resulting from the life cycle of microorganisms [25]. However, it is important to consider that factors such as contamination or impurities from the water reduction process in honey production could also contribute to sedimentation.

### 2.3. Stability Test of Honey Aromatic Water

**Figure 1:**
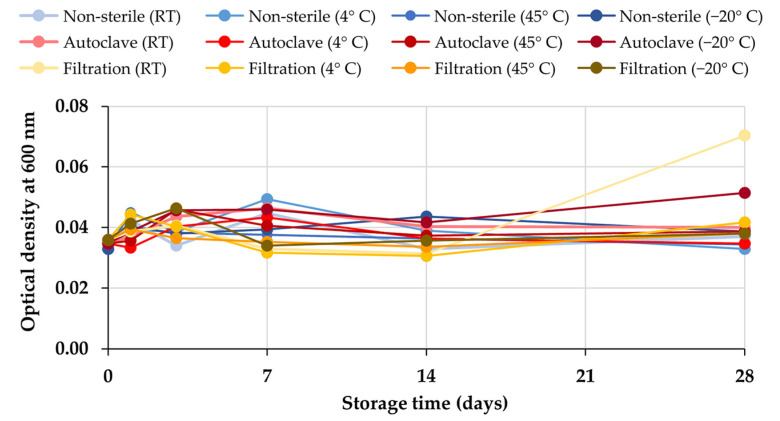
The turbidity of non-sterile and sterile honey aromatic water by autoclaving and filtration in terms of optical density detected at 600 nm.

According to the 28-day stability test, the external appearance of non-sterile and sterile honey aromatic water by filtration and autoclave are shown in Figure S2. The samples kept in all conditions for 28 days were homogeneous and transparent liquids with no color but had a slight honey odor. There was no change in the external appearance observed during the 28 days. Additionally, the pH of all samples remained at 4.0. The turbidity of each honey aromatic water sample determined by the absorbance measurement at 600 nm is shown in Figure 1. The results revealed that there was no variation in the turbidity of the sample after 28 days of storage (*p >* 0.05). Although bacterial growth can be visually observed, it is important to note that there are cases where bacterial growth may occur at levels below the visual detection threshold [26]. This can result in false negatives, where bacterial growth is present but not visually apparent. Therefore, to ensure accurate detection and avoid false negatives, it is recommended to complement visual observation with more sensitive techniques such as microbial culturing.

**Figure 2:**
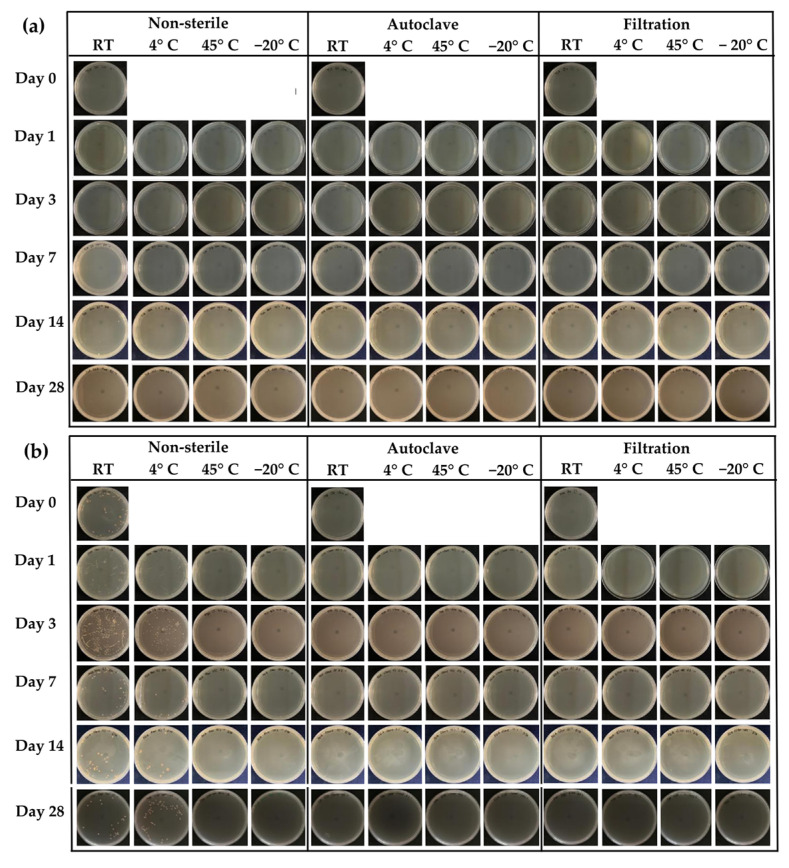
Microbiological test for the growth of bacteria when the growth medium was tryptic soy agar (TSA) (**a**) and microbiological test for the growth of molds and yeasts when the growth medium was potato dextrose agar (PDA) (**b**) of non-sterile and sterile honey aromatic water by autoclaving and filtration.

**Figure 3:**
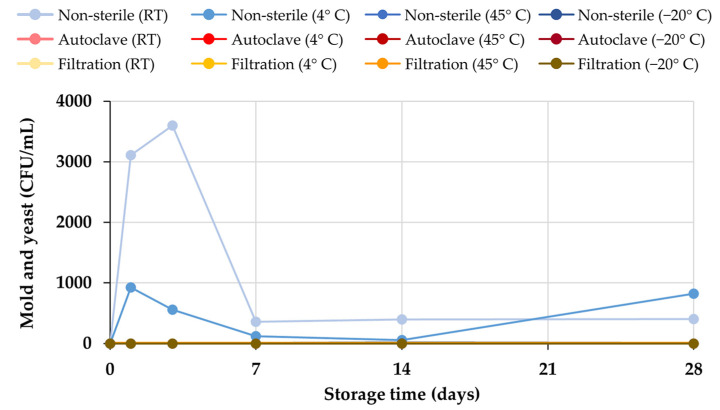
The growth of molds and yeasts in non-sterile and sterile honey aromatic water by autoclaving and filtration.

The growth of bacteria, as well as molds and yeasts, was confirmed by the microbiological test using the total plate count method. The results confirmed that there was no microbial colony growth on TSA in all storage conditions of honey aromatic water, as shown in Figure 2a, which was consistent with the results from the visual inspection and turbidity measurement. However, in contrast to the external appearance of the honey aromatic water, microbial colony growth occurred on PDA in the non-sterile water samples kept at room temperature and low temperature (4 °C), as shown in Figure 2b and Figure 3. The high water content is most likely to be accountable for the microbial growth and fermentation. Despite the fact that honey is self-preservative and resistant to microbiological growth due to its low pH, its high water content can result in microbial contamination. As a result, the water from the honey lowering water content procedure, which has a high water activity, is an excellent resource for microbes. At room temperature, the levels of microbial colonies were as high as 3110 CFU/mL after 1 day of storage and increased to 3600 CFU/mL after 3 days. After that, the microbial colony growth dramatically decreased to 360 CFU/mL after 7 days and was maintained at around 400 CFU/mL for the remaining 28 days of storage. This may be due to a lack of nutrients used by microorganisms to grow, such as fructose, glucose, sucrose, rhamnose, trehalose, etc. [27], which would lead to a transition into the log phase of microbial growth. Normally, refrigeration technologies have been used for food preservation [28]. However, the present study noted that storing the honey aromatic water in the refrigerator at 4 °C could only lower microbial colony growth. It was found that the levels of microbial colonies were 925 CFU/mL after 1 day of storage at 4 °C and decreased to 555 CFU/mL after 3 days. After that, the mold and yeast growth dramatically decreased to 120 CFU/mL after 7 days and was maintained for the remaining 28 days of storage. The growth curve of microbial colony at 4 °C followed the same pattern as storage at room temperature.

The findings highlighted that storage of the honey aromatic water at extremely low temperatures (−20 °C) and high temperatures (45 °C) could prevent the growth of bacteria, molds, and yeasts. On the other hand, sterilizations, both filtration and autoclave, were successfully used to prevent bacterial, mold, and yeast contamination.

**Figure 4:**
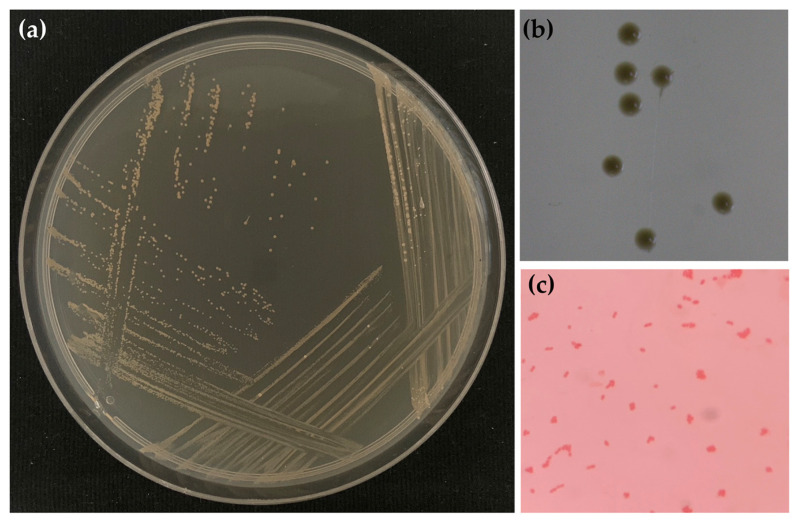
Colony morphology of microbial isolate from non-sterile honey aromatic water after the storage at room temperature for 28 days on potato dextrose agar (PDA) (**a**), under the stereo microscope with a magnitude of 2.5× (**b**), and with Gram stain under the compound light microscope with a magnitude of 100× (**c**).

To gain further insights into the growth of microbials in honey aromatic water, a microscopic examination was conducted to assess the microorganisms present in the water sample. The results as shown in Figure 4 noted that the colonies on PDA are creamy and smooth with entire margin, whereas the cells are Gram-negative, and rod-shaped. Results of 16S rRNA sequence analysis indicated that the Gram-negative isolates were identified as *Gluconacetobacter aggeris* (Table 1). It was interesting that *Gluconacetobacter aggeris* appeared on PDA and was found in non-sterile honey aromatic water storage at room temperature and 4 °C. The genus *Gluconacetobacter* is known for acetic acid bacteria that are involved in the fermentation of vinegar and can be found in sugary environments [29,30,31]. In a previous report, *Gluconacetobacter aggeris* is described as an aerobic, Gram-negative, motile bacterium isolated from the pollen of a Japanese flower [32]. As pollen is similar to all other plant tissues that are habitats for a variety of microorganisms, when honeybees collect and pack pollen, there is the possibility of microbes being present in the pollen [33], which has been noted as one of the primary sources of microbial contamination in honey and is somewhat difficult to eliminate [34].

### 2.4. Irritation Potency of Honey Aromatic Water

**Figure 5:**
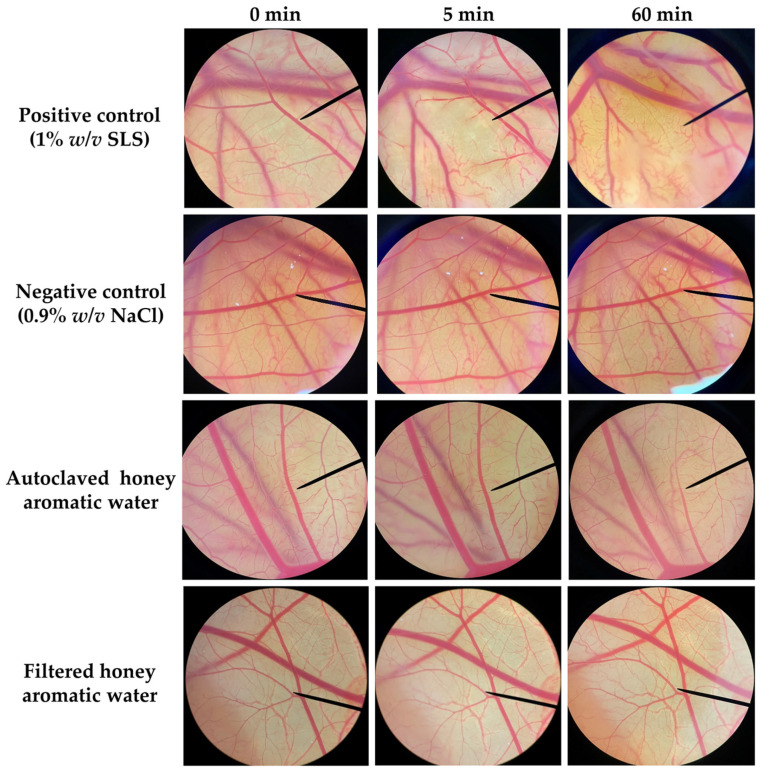
Effect of positive control (1% *w*/*v* sodium lauryl sulfate (SLS) aqueous solution), negative control (0.9% *w*/*v* sodium chloride (NaCl) aqueous solution), autoclaved honey aromatic water, and filtered honey aromatic water on the chorioallantoic membrane (CAM) at 0, 5, and 60 min.

Since honey aromatic water is a waste product from the honey industry, there are significant concerns over the safety of utilizing this water in cosmetic products. The irritation potency of honey aromatic water was evaluated by the hen’s egg test–chorioallantoic membrane (HET-CAM) assay, which was developed as an alternative to the Draize eye irritation test [35] and has been widely used for assessing the irritant potential of cosmetic ingredients designed for facial care, particularly those intended for use in the vicinity of the eye area. The HET-CAM test in the present study has been verified for its validity. An aqueous solution of 1% *w*/*v* sodium lauryl sulfate (SLS), which was used as a positive control, induced severe irritation with an irritation score (IS) of 11.81 ± 0.19, whereas a normal saline solution (0.9% *w*/*v* NaCl), which was used as a negative control, induced no irritation (Table 2). Vascular lysis and hemorrhage were observed after 5 min of exposure to the SLS, as shown in Figure 5. Consequently, more severe vascular lysis and coagulation were observed after 60 min. Contrarily, honey aromatic water from both the autoclave and the filtering process did not cause any irritation signs on the CAM, indicating that both were safe for topical application even to areas that are delicate like the mucous membrane or the region around the eyes.

### 2.5. Film-Forming Gel Base

**Figure 6:**
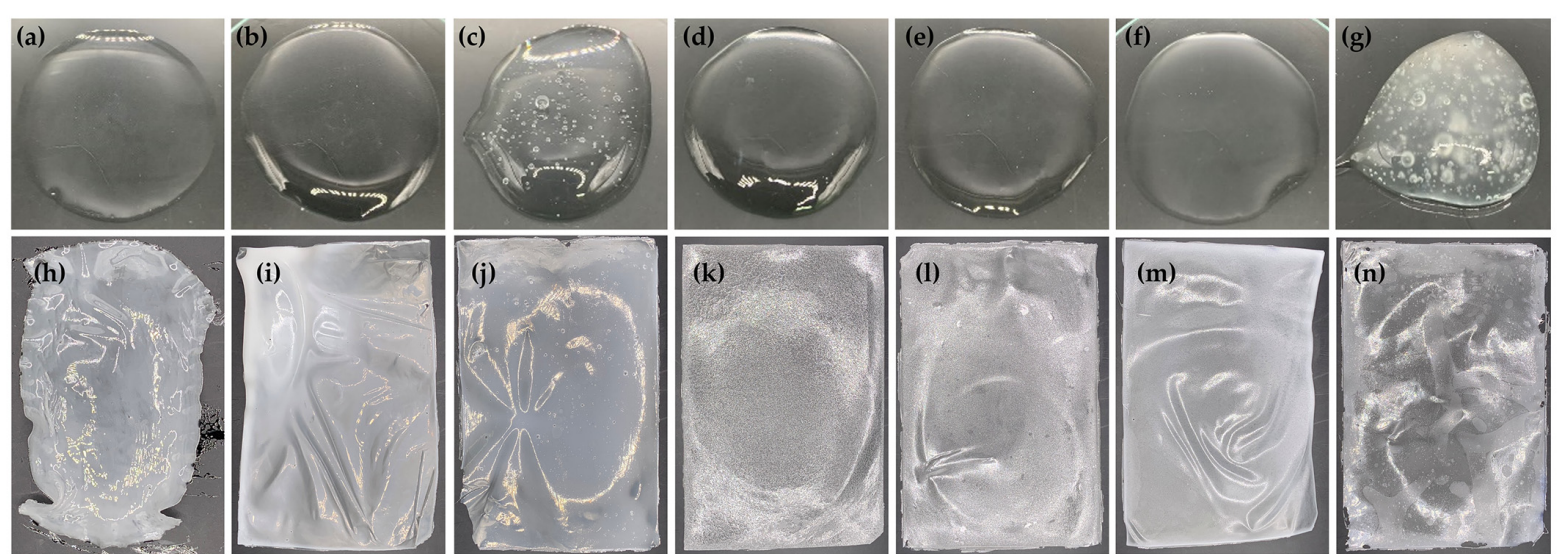
The external appearance of film-forming gel base formulation 1 containing 3% *w*/*w* PVA 117 (**a**), formulation 2 containing 3% *w*/*w* PVA 117 and 1% *w*/*w* CMC (**b**), formulation 3 containing 3% *w*/*w* PVA 117 and 2% *w*/*w* CMC (**c**), formulation 4 containing 3% *w*/*w* PVA 117 and 1% *w*/*w* HEC (**d**), formulation 5 containing 3% *w*/*w* PVA 117 and 2% *w*/*w* HEC (**e**), formulation 6 containing 3% *w*/*w* PVA 117, 0.5% *w*/*w* CMC, and 0.5% *w*/*w* HEC (**f**), and formulation 7 containing 3% *w*/*w* PVA 117, 1% *w*/*w* CMC, and 1% *w*/*w* HEC (**g**). All formulations contained 5% *w*/*w* PEG 400. The external appearance of film from film-forming gel base formulations 1 (**h**), 2 (**i**), 3 (**j**), 4 (**k**), 5 (**l**), 6 (**m**), and 7 (**n**).

In the development of film-forming gel bases, various film-forming polymers were used, including polyvinyl alcohol (PVA 117) as the main polymer, along with carboxymethyl cellulose (CMC) and hydroxyethyl cellulose (HEC). The external appearance of each film-forming gel base formulation and its film are shown in Figure 6. PVA was employed as the main polymer in the present study because it has been widely utilized for film fabrication with extraordinary properties, including biodegradability, non-carcinogenicity, high biocompatibility, ease of production, chemical resistance, and mechanical qualities [36]. Although PVA 117 could generate a transparent gel with an aesthetic appearance, as shown in Figure 6a, its film was not in shape and was difficult to peel off, as shown in Figure 6h. Therefore, CMC and HEC were also used as secondary polymers in the formulations. All film-forming gel bases were transparent gels but with different viscosities (Table 3).

Although low concentrations (1% *w*/*w*) of both CMC and HEC enhanced the viscosity of the gel in the same manner with no significant difference in viscosity, higher concentrations (2% *w*/*w*) of HEC yielded the gel with a significantly higher viscosity than that of CMC. However, air bubbles were observed in formulations with high viscosity, particularly those containing a significant amount of CMC. This observation can likely be attributed to the differences in the preparation processes. The CMC stock solution was prepared without the assistance of heat, while HEC required heating to approximately 80 °C. The heating process could have affected the viscosity of the formulation. The stock solutions of PVA and HEC reduced viscosity during the heating process and were properly mixed, resulting in a gel without bubbles after cooling down to room temperature. The spreadability of each film-forming gel showed a strong inverse relationship with its viscosity, indicating that gels with lower viscosities spread more easily. This correlation was notably pronounced in the film-forming gel composed solely of PVA-117 as its film-forming polymer, exhibiting the lowest viscosity of 0.27 ± 0.01 mPa·s with the highest spreadability of 11.5 ± 0.7 cm.

The addition of a secondary film-forming polymer to the formulation resulted in decreased spreadability and increased viscosity. The spreadability of film-forming gels containing PVA-117 in combination with secondary film-forming polymer(s) ranged from 2.9 ± 0.1 to 5.7 ± 0.2 cm. These findings align with the spreadability of previously reported peel-off gel masks containing *Achillea millefolium* designed for cosmeceutical applications, which utilized a combination of PVA and other film-forming polymers (hydroxypropyl methylcellulose, or HPMC), which exhibited spreadability ranging from 4.8 to 5.4 cm [37]. Additionally, the peel-off gel mask with PVA and gelatin yielded a spreadability in the range of 4.2 to 5.8 cm [38].

Regarding the pH of film-forming gel bases, PVA 117 alone yielded a gel with a pH of 4.5. The addition of CMC enhanced the pH to 5.5, whereas the addition of HEC had no effect on the pH. On the other hand, the addition of both CMC and HEC to the PVA 117 gel yielded a pH of 5.0. The pH, ranging from 4.5 to 5.5, was suitable for skin, as the physiological pH of the stratum corneum is 4.1–5.8 [39]. Additionally, acidic pH is beneficial to the human skin, since an acidic skin pH (4–4.5) keeps the resident bacterial flora attached to the skin, whereas an alkaline pH (8–9) promotes dispersal from the skin [40].

Not only does CMC affect pH value, but it also affects the drying time of film-forming gel bases. The addition of CMC to the gels of PVA 117 dramatically increased the drying time, whereas the addition of HEC decreased the drying time. The results from the drying time on both the glass slide and the piglet skin were consistent. However, the film-forming gel dried more quickly on pigskin skin compared to a glass slide. This could be primarily due to the disparities in the amount of gel applied. Different amounts of film-forming gel applied to the glass slide and piglet skin were due to their coverage ability on the same surface area. To ensure thorough coverage on the glass slide, a greater amount of film-forming gel was applied. In contrast, a lower amount of film-forming gel was required when applying it to piglet skin, as it could spread more uniformly. Another factor that led to the shorter drying period when applying the film-forming gel to the piglet skin was that not only was the water evaporated into the atmosphere, but the moisture from the gel was also absorbed by the piglet skin. Consequently, the gel formed a film more rapidly during the drying process. As being capable of drying quickly and requiring a short duration for the gel to dry and form the film are its desirable characteristics [41], HEC was proposed as a suitable polymer to generate the film-forming gel. However, the film-forming gel base containing HEC yielded a film with uneven texture, as shown in Figure 6k,l. In contrast, the addition of CMC generated a film with homogeneity and evenness. The combination of both CMC and HEC in PVA 117 gel reduced the limitations and disadvantages of each gelling agent. Formulation 6 was found to be a homogenous transparent gel that generated a homogeneous and evenly distributed film.

The films from each formulation were assessed for mechanical properties. Film-forming gel using PVA 117 alone was excluded from further analysis due to its unacceptable quality, as shown in Figure 6h. The tensile strength, elongation, and Young’s modulus of each film are listed in Table 3. A higher concentration of the film-forming polymers yielded a film with higher tensile strength. At the same concentration, CMC yielded a film with significantly higher tensile strength compared to HEC. Moreover, increasing the concentration of CMC possessed a more pronouncedly increased tensile strength, whereas no significant effect was found in the case of HEC. However, the mixture of PVA 117, CMC, and HEC dramatically enhanced the tensile strength of the films. As greater tensile strength indicates higher resistance to mechanical damage of the film and films with lower tensile strength tear more easily [42], a mixture of PVA 117, CMC, and HEC was suggested for a film with desirable and stronger resistance for withstanding or enduring mechanical forces. On the other hand, the individual addition of CMC and HEC had no differing effects on film elongation, but combining these two polymers and adding them to PVA 117 gel significantly increased the film elongation. The findings about the film elongation were consistent with their tensile strength since greater elongation values indicate higher tear resistance in the film layer. Similarly, Young’s modulus aligned well with other mechanical properties. In addition, the adhesion force showed a strong correlation with the elongation results, as films in formulation 3 and 4, with a very low percentage of elongation (2.86%), were unable to be peeled off and eventually pulled apart. The incorporation of royal jelly into the film-forming gel formulations resulted in reduced adhesion, making it easier to peel off. No significant difference in adhesion properties was observed among the films containing various concentrations of royal jelly in the formulation.

Formulation 7 was found to be the film-forming gel that generated films with the most resistance to mechanical damage. However, due to the unpleasant external appearance of both the gel and its film, it was excluded from further study. Formulation 6, which exhibited comparable elongation and adhesion as well as having the second highest tensile strength and Young’s modulus after Formulation 7, also had a pleasing aesthetic appearance in both the film-forming gel and the resulting film. Therefore, formulation 6 was selected for the subsequent incorporation of honey aromatic water and royal jelly.

### 2.6. Film-Forming Gel Containing Honey Aromatic Water with and without Royal Jelly

**Figure 7:**
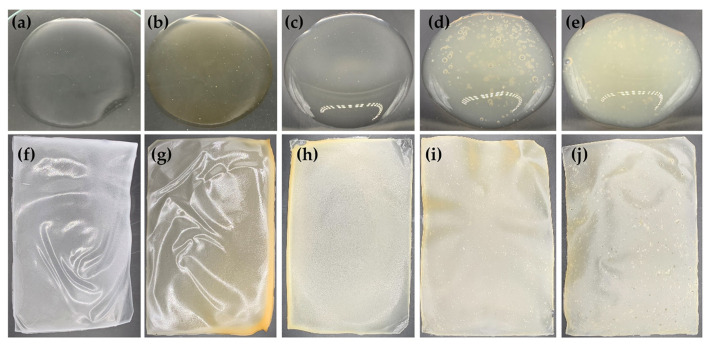
The external appearance of film-forming gel base formulation 6 containing 3% *w*/*w* PVA 117, 0.5% *w*/*w* CMC, and 0.5% *w*/*w* HEC (**a**); formulation 6A containing honey aromatic water (**b**); formulation 6R0.5 containing honey aromatic water and 0.5% *w*/*w* royal jelly (**c**); formulation 6R1 containing honey aromatic water and 1% *w*/*w* royal jelly (**d**); and formulation 6R2 containing honey aromatic water and 2% *w*/*w* royal jelly (**e**). The external appearance of film from film-forming gel formulation 6 (**f**), 6A (**g**), 6R0.5 (**h**), 6R1 (**i**), and 6R2 (**j**).

Regarding the aesthetic characteristics of both the film-forming gel and its film, formulation 6, containing 3% *w*/*w* PVA 117, 0.5% *w*/*w* CMC, and 0.5% *w*/*w* HEC, along with 5% *w*/*w* PEG 400, was selected for the incorporation of honey aromatic water and royal jelly. The external appearance of the film-forming gel containing honey aromatic water with and without royal jelly and their films are shown in Figure 7 and Table 4. After incorporating the honey aromatic water, the gel remained transparent, but the color turned pale yellow. Similarly, the films formed from film-forming gel using DI water and honey aromatic water as vehicles had the same characteristics except in terms of color. As the honey aromatic water had a low pH of around 4.0, its formulation had a lower pH (Table 4). However, the viscosity significantly decreased from 1.26 ± 0.01 mPa·s to 0.91 ± 0.02 mPa·s after incorporation of the honey aromatic water (Table 4). The likely explanation could be due to both PVA 117 and CMC, which are incompatible with strong acids. PVA has been known to decompose in strong acids and soften in weak acids [43]. Similarly, CMC is incompatible with strongly acidic solutions and precipitation may occur at pHs lower than 2 [44]. In contrast, HEC has good tolerance for dissolved electrolytes [45]. Nonetheless, it is essential to note that the pH level of formulation 6A is 4.5, suggesting that it is weakly acidic. In view of this finding, it is critical to emphasize that all of the polymers used in this formulation are considered acceptable and appropriate.

Similar to the film-forming gel base, the viscosity and gel spreadability of the film-forming gel containing honey aromatic water with and without riyal jelly were inversely correlated (Table 4). The formulation containing 2% *w*/*w* of royal jelly exhibited higher viscosity than those containing a lower concentration. However, its spreadability was not significantly different from the others, except for the formulation containing 0.5% *w*/*w* of royal jelly. Therefore, the formulation of 2% *w*/*w* royal jelly was suitable for further topical applications, as it combines an aesthetically pleasing external appearance characterized by high viscosity while also demonstrating excellent spreadability, which is a requirement for ideal gel formulation with therapeutic effectiveness [46].

In addition to its impact on pH and viscosity, honey aromatic water also reduced the drying time of the gel. The likely explanation could be due to the volatile components in the honey aromatic water that help the solvent evaporate faster than the aqueous solution. The drying time on both the glass slide and the piglet skin showed a similar trend. However, in the case of piglet skin, the drying time significantly decreased in the formulation with royal jelly. Nevertheless, the film-forming gels with royal jelly accelerated drying when applied on the piglet skin. This phenomenon could be attributed to its efficient absorption into the skin, facilitated by the properties of royal jelly as an emulsion of proteins, sugars, lipids, and other identified water-soluble compounds [47,48]. However, it was found that the drying time lengthened once the royal jelly reached a particular concentration. This could potentially be attributed to surpassing the saturation point of the absorption of moisture into the piglet skin. However, the drying time of the film-forming gel containing honey aromatic water and 2% *w*/*w* of royal jelly was 18.4 ± 1.0 min, which was not different from the formulation without royal jelly or without both royal jelly and honey aromatic water. The drying time of these film-forming gels makes them appropriate for use as peel-off masks, and the results were consistent with those of other investigations. A previous study reported that peel-off gel masks containing the ethanolic extract of *Achillea millefolium* and PVA in concentrations ranging from 7% to 10% *w*/*w* dried in 27 to 31 min [37]. On the other hand, the mixture of PVA with other film-forming polymers resulted in different drying times of around 14–19 min [49].

Besides its effect on the film-forming gels, honey aromatic water had a significant effect on the mechanical properties of the film as shown in Table 4. It was noted that the honey aromatic water reduced the tensile strength, elongation, and Young’s modulus of the films. A likely explanation could be due to the acidity of the honey aromatic water. As honey aromatic water is a by-product derived from the distillation of honey, its composition may include many organic acids commonly found in honey, such as acetic, citric, formic, fumaric, D-gluconic, D-glucuronic, glutaric, lactic, L-malic, oxalic, propionic, D-quinic, L-tartaric, succinic acid, etc. [19,20,21,22,23]. Although acids are known as crosslinking agents that yield an elastic gel with higher mechanical properties, the acid could play a role as a plasticizer and reduce the interactions among the macromolecules at high concentrations, resulting in a decrease in mechanical properties [50]. A previous study reported that various acids have been used as crosslinking agents, e.g., citric acid, fumaric acid, and malic acid [51]. Additionally, oxalic acid has been reported to show a cross-linking reaction for PVA via the formation of an ester bond between the hydroxyl groups of the PVA chain and the carboxylic group of oxalic acid [52]. A greater oxalic acid concentration produced a film with a higher tensile strength and Young’s modulus; however, once the oxalic acid concentration exceeded 10% *w*/*w*, both tensile strength and Young’s modulus drastically decreased [53]. In addition, CMC and HEC, which are cellulose derivatives, have been reported to form a gel when mixed with acid, but could be degraded slowly in strong acids [51,54]. The findings from this study highlighted that using honey aromatic water instead of DI water in the film-forming gel formulation could reduce the mechanical properties of the resulting films. The results were in line with a previous study that reported that the elongation of a PVA film dramatically decreased in the presence of oxalic acid [53].

Royal jelly, produced by the cephalic glands of nurse bees, possesses a complex composition consisting of various elements and encompasses water, proteins, lipids, carbohydrates, amino acids, mineral salts, vitamins, enzymes, hormones, oligo-elements, and natural antibiotics [55]. A previous study reported that the incorporation of royal jelly into an emulsion did not affect formulation stability but helped enhance skin absorption without leaving a greasy film. The suggested concentration of royal jelly in the formulation was between 0.5% and 1% since it exhibited moisturizing properties [8]. In the present study, up to 2% *w*/*w* of royal jelly was incorporated into the film-forming gels. It was noted that the addition of royal jelly also affected the film-forming gels. Higher concentrations of royal jelly led the formulation to become more viscous and turn turbid due to the characteristic of royal jelly, which imparts a milky appearance. However, the native acidic pH of royal jelly [56] had no effect on the pH of the formulations since its pH was around 4.0, which was equivalent to the honey aromatic water. In addition, it was observed that the drying time was somewhat extended due to the presence of royal jelly. A likely explanation could be attributed to the unique properties of royal jelly, which has a relatively higher viscosity and moisture content. On the other hand, the addition of royal jelly could enhance the mechanical properties of the films, especially in terms of tensile strength and elongation. However, the elongation of the film was found to be dramatically decreased at the concentration of 2% *w*/*w* royal jelly. This could be due to the air bubbles in the film, which make it tear apart more easily in the elongation test. Nevertheless, the peel adhesion test revealed that films from all formulations could be easily peeled off, particularly after the addition of honey aromatic water. Conversely, the addition of royal jelly increased the adhesion force but did not differ significantly from its gel base.

**Figure 8:**
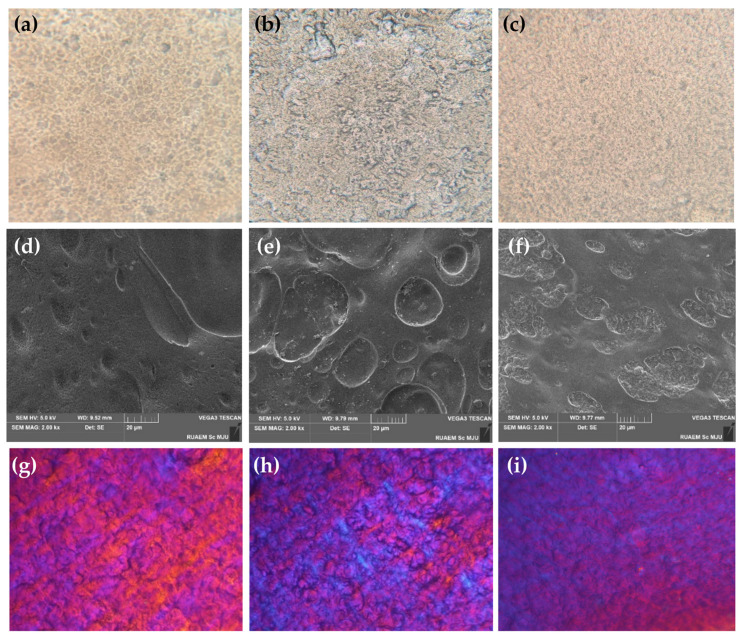
The morphology under the compound light microscope set at the magnitude of 10× of the films from film-forming gel formulation 6 (**a**), formulation 6A (**b**), and formulation 6R2 (**c**); under the field emission scanning electron microscopy set at the magnitude of 2k× of the films from film-forming gel formulation 6 (**d**), formulation 6A (**e**), and formulation 6R2 (**f**); and under the polarizing light microscope set at the magnitude of 10× of the films from film-forming gel formulation 6 (**g**), formulation 6A (**h**), and formulation 6R2 (**i**).

The microstructures of film-forming gel containing honey aromatic water with and without 2% *w*/*w* royal jelly are shown in Figure 8. Under the compound light microscope, the film with honey aromatic water was found to be the most uneven, as shown in Figure 8b. Larger irregular air gaps could be observed all over the film. This was consistent with its external appearance, showing that the film was the most translucent compared to the others, which were more opaque. In contrast, a film derived from a gel comprising both honey aromatic water and 2% *w*/*w* royal jelly, as shown in Figure 8c, displayed the most densely packed texture, appearing notably uniform and aligning with its highest degree of opaqueness among the samples. The SEM micrographs with the magnitude of 2kx, as shown in Figure 8e, were used to confirm the larger irregular air gaps in the film from the gel containing honey aromatic water. However, under a polarized light microscope, all films exhibited birefringent textures, confirming their optical anisotropy and the presence of organized structures [57].

**Figure 9:**
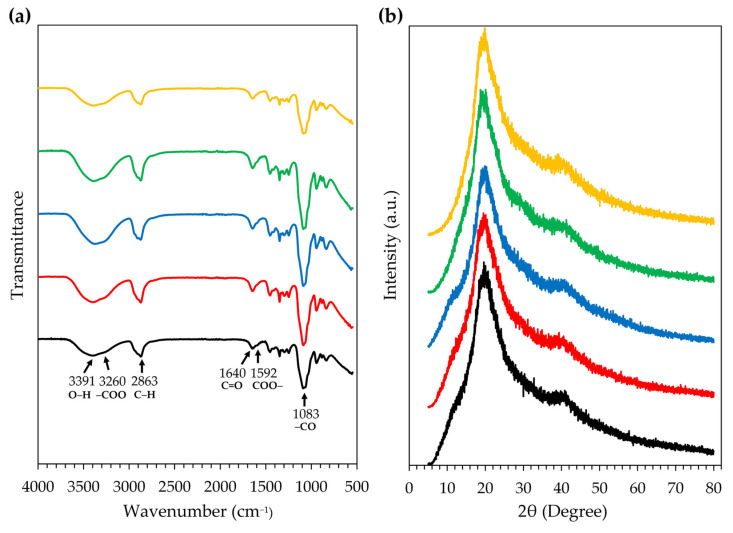
FT-IR spectra (**a**) and XRD spectra (**b**) of the films from the film-forming gel base (black), film-forming gel containing honey aromatic water (red), and film-forming gel containing honey aromatic water and royal jelly at the concentration of 0.5% *w*/*w* (blue), 1% *w*/*w* (green), and 2% *w*/*w* (yellow).

The FT-IR technique is employed to assess and identify the chemical composition of substances by measuring their absorption of infrared light, proving particularly effective for identifying functional groups, detecting chemical structures, and investigating molecular vibrations. All film samples exhibited exactly the same pattern of FT-IR spectra as shown in Figure 9a. A broad band from 3700–3100 cm^−1^ (a maximum of 3391 cm^−1^) corresponded to the O–H stretching vibration of intermolecular bonded alcohol, which was found in both PVA and HEC [36,58]. The broad absorption band of CMC at around 3260 cm^−^^1^ due to the stretching frequency of the –COO group overlapped with the –OH stretching region [59]. The medium absorption band in the region 3000–2840 cm^−^^1^ (a maximum of 2863 cm^−^^1^) was a result of C–H stretching vibration of alkane [36,58,59]. The strong peaks at 1083 cm^−^^1^ corresponded to the C–O stretching vibrations [36]. Numerous complicated peaks in the low wavenumber region were observed as follows: 1640 cm^−^^1^ (C=O stretching vibration) [60], 1592 cm^−^^1^ (antisymmetric vibration of COO–) [59], 1240 cm^−^^1^ (C–H wagging vibrations) [36], 1062 cm^−^^1^ (C–O–C stretching vibration in the glucopyranose) [58], 1060 cm^−^^1^ (CH-O-CH_2_ stretching) [59], 1026 cm^−^^1^ (C–C–C stretching vibration) [36], and 887 cm^−^^1^ (β-(1,4) glycoside linkage) [58]. As there was no difference among the FT-IR spectra of all film samples, it could be concluded that both honey aromatic water and royal jelly had no effect on the functional groups and chemical structures of the film-forming polymers.

XRD is an analytical method that utilizes X-rays to examine and identify the crystal structure of a sample. The XRD spectra of films formed using film-forming gels, as shown in Figure 9b, showed the same pattern with a characteristic crystalline peak at 2θ = 19.2° and 40.5°. The results were in line with a previous study that reported the crystalline peaks of PVA at 2θ = 19.5° and 40.8° [36,61,62], CMC at 2θ = 20° [63], and HEC at 2θ~20.27° [61]. The findings indicated that the addition of honey aromatic water and royal jelly had no effect on the crystallinity of the film.

## 3. Conclusions

Honey aromatic water obtained from the water reduction process of honey could be beneficial for skin care applications due to its mild acidity and slight honey odor. However, microbial contamination was detected when it was kept at an ambient temperature. Storage at extremely low temperatures (−20 °C) and high temperatures (45 °C) could prevent microbial growth. Sterilization processes, including both filtration and autoclaving, were suggested to maintain the sterility of honey aromatic water before the development of any formulations. In the present study, a film-forming gel was successfully developed using a mixture of PVA 117, CMC, and HEC. Furthermore, the successful incorporation of honey aromatic water and royal jelly into the film-forming gel matrix was achieved. The findings highlighted that honey aromatic water, with a mild acidity at a pH of 4.0, resulted in a decrease in gel viscosity and the mechanical properties of its film. Interestingly, the drying time was dramatically decreased from 73.3 ± 1.2 min to 47.7 ± 0.6 min when applied on the glass slide and from 19.4 ± 0.6 min to 17.4 ± 0.1 min when applied on the piglet skin, which would be more desirable for using as a peel-off mask. Furthermore, royal jelly was successfully incorporated into the film-forming gel of honey aromatic water, and it was found to enhance the viscosity of the gels and improve the mechanical properties of the film, with a significant decrease in the drying time when low concentrations were incorporated. The macroscopic investigations show irregular air gaps in the film from the gel containing honey aromatic water. However, this issue could be solved by the incorporation of royal jelly. On the other hand, both honey aromatic water and royal jelly had no effect on the chemical and crystal structure of the films. Therefore, it can be concluded that the film-forming gels containing honey aromatic water and royal jelly are suitable for using as a peel-off mask with the aesthetic characteristics of both the gels and their films. Further clinical study in human volunteers for the efficacy on skin is suggested.

## 4. Materials and Methods

### 4.1. Honey Aromatic Water

Honey aromatic water was obtained from Ban Me Thuot Honey Bee JSC, Dak Lak, Vietnam and kept at 4 °C in an airtight container until further usage.

### 4.2. Chemical Materials

Royal jelly was purchased from Chiangmai Healthy Product Co., Ltd. (Chiang Mai, Thailand). Potato dextrose broth and tryptic soya broth were purchased from Sigma Aldrich (St Louis, MO, USA). Polyvinyl alcohol (PVA 117) with a viscosity range of 22–30 mPa·s and hydroxyethyl cellulose (HEC) with a viscosity of 2000 mPa·s were purchased from Chanjao Longevity Co., Ltd. in Bangkok, Thailand. Carboxymethyl cellulose (CMC) was purchased from Dow Chemical Company (Midland, MI, USA). Polyethylene glycol 400 (PEG 400) was purchased from Namsiang Co., Ltd. (Bangkok, Thailand).

### 4.3. Sterilization Process of Honey Aromatic Water

#### 4.3.1. Filtration

Honey aromatic water was filtered through a nylon syringe filter with a pore size of 0.22 microns. The sterile water was stored in sterilized bottles until further experiments.

#### 4.3.2. Autoclave

Honey aromatic water was sterilized by steam sterilization using an autoclave. The temperature was set at 121 °C with a steam pressure of 15 pounds per square inch. The honey aromatic water was autoclaved for 30 min. The sterile water was stored in sterilized bottles until further experiments.

### 4.4. Characterization of Honey Aromatic Water

Honey aromatic water was characterized for its external appearance in terms of color, odor, and clarity. The turbidity of honey aromatic water was investigated using a 96-well microplate reader set at 600 nm [64]. In addition, the pH of honey aromatic water was investigated using a universal pH paper. The pH measurement was performed in triplicate.

### 4.5. Stability Test of Honey Aromatic Water

#### 4.5.1. Storage Conditions

Honey aromatic water, both non-sterile and sterile, was kept in light-protected sterilized bottles and kept at various conditions, including room temperature, 20 °C, 4 °C, and 45 °C, for 28 days. The stability of honey aromatic water was examined on days 0, 1, 3, 7, 14, and 28 in terms of external characteristics, turbidity, pH, and microbial test.

#### 4.5.2. Characterizations

Honey aromatic water kept in various conditions was examined for its external characteristics, turbidity, and pH as mentioned above.

#### 4.5.3. Microbial Counts Analysis

Honey aromatic water kept in various storage conditions was examined for the microbial contamination of bacteria and fungi using the total plate count method. Tryptic soy agar (TSA) was used to test for bacterial contamination, whereas potato dextrose agar (PDA) was used to test for fungal contamination. After applying the tested samples to the culture medium, TSA was incubated at 37 °C for 24 h, whereas PDA was incubated at 30 °C for 72 h. The number of microbial colonies was recorded and calculated as colony-forming units per mL of honey aromatic water (CFU/mL). The microbial colony that appeared on the culture media was identified morphologically using the Gram stain. A pure microbial culture from PDA was sent to Macrogen Company in Korea for 16S ribosomal RNA (rRNA) sequence analysis.

### 4.6. Irritation Test by Hen’s Egg Test–Chorioallantoic Membrane (HET-CAM) Assay

The honey aromatic water, both after autoclaving or filtration, was evaluated for its skin irritation potency using HET-CAM assay [65]. Hen’s eggs with an age of 7 days, which were in the early embryonic growth section (between 3 and 7 days), do not yet represent the midpoint of the incubation period, and do not need any ethical committee approval, were used in the present study [66]. The hen’s eggs were incubated in an automated rotating egg incubator (Nanchang Howard Technology Co., Ltd., Jiangxi, China) set at 37.5 ± 0.5 °C and 62.5 ± 7.5% relative humidity. The shell of each hen’s egg was opened by a rotating cutting blade attached to a Marathon-3 Champion dental micromotor (Saeyang, Daegu, Republic of Korea) in the area above the air cell. The inner membrane directly in contact with the CAM was moistened with 0.9% *w*/*v* NaCl aqueous solution and carefully removed using forceps. The appearance of the CAM was recorded as a photo under a stereo microscope (Olympus, Tokyo, Japan). Consequently, 30 µL of each honey aromatic water sample was carefully dropped onto the CAM. Once the samples were applied, we started to record the time. Irritation signs, including hemorrhage, vascular lysis, and coagulation, were observed under a stereo microscope (Olympus, Tokyo, Japan) for 5 min. The irritation score (IS) was then calculated using the following equation:

IS = [(301 − *th*) × 5]/300 + [(301 − *tl*) × 7]/300 + [(301 − *tc*) × 9]/300, (1)

where *th* is the time in seconds of the first hemorrhage observed, *tl* is the time in seconds of the first vascular lysis observed, and *tc* is the time in seconds of the first coagulation observed. The irritation potency was classified as non-irritation (IS = 0.0–0.9), slight irritation (IS = 1.0–4.9), moderate irritation (IS = 5.0–8.9), and severe irritation (IS = 9.0–21.0) [65]. Lastly, the CAMs were observed under a stereo microscope (Olympus, Japan) again after 60 min. Sodium lauryl sulfate aqueous solution (1% *w*/*v*) was used as a positive control, whereas NaCl aqueous solution (0.9% *w*/*v*) was used as a negative control. All experiments were performed in duplicate.

### 4.7. Development of Film-Forming Gel Base

#### 4.7.1. Preparation of Film-Forming Gel Base

Film-forming gel base was developed using various types and concentrations of film-forming polymers, including PVA 117, CMC, and HEC. PEG 400 was used as a plasticizer in all formulations. The formulations of film-forming gel bases are demonstrated in Table 5. To prepare the film-forming gel base, each polymer was separately dispersed in DI water. PVA 117 was used as the main film-forming polymer in all formulations. In the preparation of the PVA 117 and HEC stock solutions, each film-forming polymer was gradually dispersed in DI water, which was placed on a multiple heating magnetic stirrer (AM4, Velp Scientifica, Usmate Velate, Italy) set at 1500 rpm and heated at 80 °C for 15–30 min until the mixture was homogeneous. In the case of CMC, the stock solution was prepared using the same method at ambient temperature without heating. Finally, the stock solutions of all film-forming polymers were combined together, and PEG 400 aqueous solution was then added and mixed until homogeneous using a multiple heating magnetic stirrer (AM4, Velp Scientifica, Italy) set at 1500 rpm and heated at 80 °C. After the mixture was cooled down to around 50 °C, the film-forming gel base was kept in sealed aluminum foil packaging.

#### 4.7.2. Characterizations of Film-Forming Gel Base

- External appearances

The external appearance of the film-forming gel base was characterized in terms of transparency, color, and homogeneity by visual and organoleptic inspections.

- Viscosity measurement

The viscosity of the film-forming gel base was measured using a Brookfield R/S rheometer (P25, Brookfield, WI, USA) set at 25 °C. The film-forming gel base was immediately measured once the packaging was open, and the measurement was finished within 5 min to prevent the film from forming. Each formulation was placed on the plate, and the rotating disc was then lowered, initiating the measurement. Each film-forming gel base formulation was separately measured three times.

- Spreadability measurement

The spreadability of the film-forming gel base was measured using the method of Bachhav and Patravale (2009) [67], with some modifications, as shown in Figure S3. In brief, a total of 0.18 g of film-forming gel base was placed in the middle of a glass plate with a size of 20 × 5 cm. Subsequently, another glass plate of identical dimensions, weighing 46.68 g, was placed on top. Both glass plates were positioned on a TA.XT PLUS texture analyzer (Stable Micro Systems Ltd., Godalming, UK), which was equipped with a 1 kg load cell. Afterward, a P/10 cylindrical Perspex (Lucite International Ltd., Queens Gate, UK) probe was lowered onto the surface of an upper glass plate with a constant speed of 1 mm/s. After 30 s of contact, the probe was moved upwards at a constant speed of 1 mm/s. The diameter of the gel spread was measured and reported in cm. Each film-forming gel base formulation was separately measured three times.

- pH measurement

The pH of each film-forming gel base formulation was measured using a universal pH paper. Each film-forming gel base formulation was separately measured three times.

- Evaporation time measurement

Evaporation time is the time required for the film-forming gel base to dry. The evaporation time of each film-forming gel base was investigated by applying the film-forming gels on both glass slides and piglet skin. The piglet skin was obtained from the flank area of stillborn piglets which naturally died before birth. In this case, the ethical issues associated with using animal skin can be bypassed [68]. Prior to the study, the piglet skin was carefully shaved with a razor to remove the hair. The skin was then cleaned and rinsed with normal saline solution (0.9% *w*/*v* NaCl), blotted dry with tissue paper, and blow-dried. The thickness of the skin, measured by a micrometer, was 0.065 mm. In the measurement of evaporation time, an adequate amount of film-forming gel that could cover an area of 1.8 × 1.8 cm was applied to each surface. In brief, 50 mg of the film-forming gel was applied to a glass slide, whereas the amount applied to the piglet skin was 20 mg. Immediately, after applying each film-forming gel, the timer started. On the other hand, in the measurement of evaporation time on the piglet skin, 20 mg of each film-forming gel was applied to an area of 1.8 × 1.8 cm and the timer started. The formulation was visually observed to confirm that the film was completely dry by weighing the glass slide with the formulation. The drying time was recorded once the weight of the glass slide with the formulation remained constant. Each film-forming gel base formulation was separately evaluated for evaporation time three times.

#### 4.7.3. Characterizations of the Film from Film-Forming Gel Base

To prepare the film from the film-forming gel base, 10 g of each film-forming gel was applied to a plastic plate (8.5 × 12.8 cm) and left at ambient temperature for the specified evaporation time to ensure that the film was dry. Subsequently, the films were removed from the glass slide and subjected to characterizations.

- External appearances and film thickness

The film from the film-forming gel base was characterized for its external appearance in terms of transparency, color, and homogeneity by visual and organoleptic inspections. The thickness of each film was measured using a micrometer.

- Texture analysis

Tensile strength data of the films from the film-forming gels were obtained using a TA.XT PLUS texture analyzer (Stable Micro Systems Ltd., Godalming, UK). Each film with a uniform thickness of 1.00 ± 0.1 mm and dimensions of 7.0 × 2.0 cm was carefully clamped between the tensile grip probes (A/TG), followed by stretching at the crosshead capacity of 2 mm/min with an initial distance of 30 mm in a 5 N–load cell. The pre-test speed was set at 2 mm/s, the test speed at 2 mm/s, the post-test speed was set at 10 mm/s, the trigger force was 5× *g*, and the test distance was 250 mm. The curves of force (N) as a function of distance (mm) were plotted by the Texture Expert Exceed 2.64 software (Stable Micro Systems Ltd., Godalming, UK). Tensile strength was calculated using the following equation:

Tensile strength = *PF*/*A*, (2)

where *PF* is the peak positive force and *A* is the transverse-sectional area the force is acting on. Additionally, elongation at break was calculated using the following equation:

Elongation (%) = 100 × (*L*/*L*_0_ − 1), (3)

where *L* is the length when films break and *L*_0_ is the original length. On the other hand, Young’s modulus was calculated from the slope of the stress and strain curve in the first linear part of the curve. All measurements were conducted in triplicate.

- Peel adhesion test

The peel test is popular for adhesion measurements by evaluating the force required to peel a film off from the attached surface [69]. The 90° peel adhesion test was conducted following the method of Qi and Sun (2010) [70], with the modifications shown in Figure S4. Prior to the investigation, a total of 0.8 g of each film-forming gel was thoroughly applied on a glass slide with a size of 7.5 × 2.5 cm and left at an ambient temperature for 30 min to allow the gel to completely dry and the film to be formed. One side of the film was peeled off from the glass slide, doubled back at a 90° angle, and attached to the A/TG tensile grip probe, while the glass slide was fixed on the heavy-duty platform (HDP/90) of a TA.XT PLUS texture analyzer (Stable Micro Systems Ltd., Godalming, UK). The distance between the HDP/90 and A/TG tensile grip probe was initially set at 50 mm, with the probe moving upward at a constant speed of 1 mm/s with a force of 0.049 N. Data related to adhesion energy were collected and reported in units of N/mm after the film was peeled, and the reported values are the average of the three replications for each treatment.

### 4.8. Development of Film-Forming Gel Containing Honey Aromatic Water with and without Royal Jelly

#### 4.8.1. Preparation of Film-Forming Gel Base Containing Honey Aromatic Water with and without Royal Jelly

The film-forming gel base with an aesthetic appearance and suitable characteristics was selected for the incorporation of honey aromatic water, which was used in place of DI water in the formulation. Furthermore, royal jelly was added to the film-forming gel containing honey aromatic water in various concentrations, ranging from 0.5 to 2% *w*/*w*. In the preparation process, the film-forming gel containing honey aromatic water was firstly prepared as described above. After the formulation cooled down to about 40 °C, royal jelly was added and continuously mixed until homogeneous using a multiple-heating magnetic stirrer (AM4, Velp Scientifica, Italy) set at 1500 rpm. The formulations are shown in Table 6.

#### 4.8.2. Characterizations of Film-Forming Gel Containing Honey Aromatic Water with and without Royal Jelly

The film-forming gel base was characterized for its external appearance in terms of transparency, color, and homogeneity by visual and organoleptic inspections. In addition, the formulation was characterized for its viscosity, spreadability, pH, and evaporation time, as mentioned previously.

#### 4.8.3. Characterizations of the Film from Film-Forming Gel Containing Honey Aromatic Water with and without Royal Jelly

To prepare the film from the film-forming gel base containing honey aromatic water with and without royal jelly, 10 g of each formulation was applied to a plastic plate (8.5 × 12.8 cm) and left at ambient temperature for the specified evaporation time to ensure that the film was dry. Subsequently, the films were removed from the glass slide and subjected to analyses.

- External appearances and film thickness

The film from the film-forming gel containing honey aromatic water with and without royal jelly was characterized for its external appearance in terms of transparency, color, and homogeneity by visual and organoleptic inspections. The thickness of each film was measured using a micrometer.

- Micro-structural analysis

The film from the film-forming gel containing honey aromatic water with and without royal jelly was characterized for its micro-structural details using a compound light microscope (Zeiss stemi 508, Carl Zeiss, Jena, Germany), polarizing light microscope (Motic-model no. BA310 Pol, Carlsbad, CA, USA), and field emission scanning electron microscope (FE-SEM; CLARA, TESCAN, Brno, Czech Republic). In the case of a stereo and polarizing light microscope, the film (4 × 4 cm) mounted on a glass slide was observed under the microscope with a magnitude of 10×. On the other hand, the film was kept in a desiccant for 30 min to reduce its moisture to less than 6%, then cut to 1 × 1 cm and attached to the carbon adhesive tape. The film was then attached to the sample platform and coated with gold using the CCU-010 high vacuum sputter and carbon coater (Safematic CCU-010, Zizers, Switzerland) to a thickness of 8 nm to make the sample electrically conductive at high vacuum conditions and reduce damage from the heat of the electron beam hitting the film. The film sample was observed under the microscope with a magnitude of 2k×.

- Texture analysis

Tensile strength data of the films from the film-forming gel containing honey aromatic water with and without royal jelly were obtained using a TA.XT PLUS texture analyzer (Stable Micro Systems Ltd., Godalming, UK) as previously described. All measurements were conducted in triplicate.

- Fourier transform infrared spectroscopy (FT-IR)

The chemical reactions, molecular structures, and compositions of the film from the film-forming gel containing honey aromatic water with and without royal jelly were investigated using an FT-IR spectrometer equipped with the single reflection diamond ATR module (ALPHA II, Bruker, Karlsruhe, Germany). Firstly, we placed the 2 × 2 cm film on the diamond crystal. Then, we lowered the pressure tip to press the film into close contact with the diamond crystal so the IR beam could transmit the sample above the top of the diamond crystal. The IR spectrum was then scanned and recorded with a wavenumber in the 400–4000 cm^−^^1^ range. The FTIR spectra were plotted with transmittance on the Y-axis and the wavenumber (cm^−1^) on the X-axis.

- X-ray diffraction (XRD) analysis

The crystal phase and structure of the film from the film-forming gel containing honey aromatic water with and without royal jelly were analyzed by an XRD (D2 PHASER, Bruker, Karlsruhe, Germany) with X-ray source (Cu K-alpha, 1.54 Angstrom of wavelength) operating at 30 kV and 10 mA. Firstly, we placed the 2 × 2 cm film on the XRD holder (Specimen rings, 25 mm). Then, we placed the holder onto the sample plate in the XRD chamber and started scanning at 2-theta from 5–80° with a time per step of 0.2°·s^−1^. The resulting spectra were plotted with intensity (a.u.) values on the Y-axis and the 2-theta angle (degrees) on the X-axis.

## Figures and Tables

**Table 1:** Results from the BLAST analysis of the 16S rRNA gene sequence for bacterial identification.

Isolate Code	Accession NO.	Size of Nucleic Acid (bp)	Closet Match Species	Identity (%)
HAW01	NR_114382.1	1414	*Gluconacetobacter aggeris*	100

**Table 2:** Irritation score (IS) from the HET-CAM assay.

Samples	Irritation Score	Irritation Potency
Positive control (1% *w*/*v* SLS)	11.81 ± 0.19	Severe irritation
Negative control (0.9% *w*/*v* NaCl)	0.00 ± 0.00	No irritation
Autoclaved honey aromatic water	0.00 ± 0.00	No irritation
Filtered honey aromatic water	0.00 ± 0.00	No irritation

Note: SLS = sodium lauryl sulfate, NaCl = sodium chloride.

**Table 3:** Characteristics of film-forming gel base.

Parameters	Film-Forming Gel Base Formulations						
	1	2	3	4	5	6	7
**Film-forming gel**							
Viscosity (mPa·s)	0.27 ± 0.01 ^f^	0.75 ± 0.02 ^e^	1.91 ± 0.08 ^c^	0.81 ± 0.03 ^e^	2.24 ± 0.13 ^b^	1.26 ± 0.01 ^d^	4.43 ± 0.04 ^a^
Spreadability (cm)	11.5 ± 0.7 ^a^	4.7 ± 0.5 ^c^	2.9 ± 0.1 ^d^	5.7 ± 0.2 ^b^	3.4 ± 0.2 ^d^	4.5 ± 0.1 ^c^	3.1 ± 0.1 ^d^
pH	4.5	5.5	5.5	4.5	4.5	5.0	5.0
Drying time (min)							
on glass slide	85.3 ± 0.6 ^c^	88.3 ± 0.6 ^b^	74.3 ± 1.6 ^a^	59.3 ± 1.2 ^f^	62.7 ± 0.6 ^e^	73.3 ± 1.2 ^d^	73.7 ± 0.6 ^d^
on piglet skin	22.3 ± 1.1 ^b^	22.6 ± 0.6 ^b^	25.3 ± 1.3 ^a^	19.5 ± 1.1 ^c,d^	19.6 ± 0.6 ^c,d^	19.4 ± 0. 6 ^d^	21.9 ± 0.3 ^b,c^
**Film**							
Tensile strength (N/mm^2^)	ND	5.52 ± 1.69 ^c^	13.52 ± 0.54 ^a,b^	3.18 ± 0.24 ^c^	4.29 ± 0.11 ^c^	11.05 ± 2.54 ^b^	15.69 ± 0.89 ^a^
Elongation (%)	ND	4.29 ± 0.00 ^c^	2.86 ± 0.00 ^c^	2.86 ± 0.00 ^c^	5.00 ± 1.01 ^b,c^	9.29 ± 1.01 ^a^	7.14 ± 2.02 ^a,b^
Young’s modulus (kPa)	ND	1.29 ± 0.39 ^c^	4.73 ± 0.19 ^a^	1.11 ± 0.08 ^c^	0.87 ± 0.15 ^c^	1.18 ± 0.14 ^c^	2.27 ± 0.52 ^b^
Adhesion (N/mm)	ND	53.5 ± 14.5 ^a^	N/A	N/A	20.8 ± 1.9 ^b^	19.8 ± 1.0 ^b^	30.1 ± 7.6 ^b^

NOTE: Formulation 1 containing 3% *w*/*w* PVA 117, formulation 2 containing 3% *w*/*w* PVA 117 and 1% *w*/*w* CMC, formulation 3 containing 3% *w*/*w* PVA 117 and 2% *w*/*w* CMC, formulation 4 containing 3% *w*/*w* PVA 117 and 1% *w*/*w* HEC, formulation 5 containing 3% *w*/*w* PVA 117 and 2% *w*/*w* HEC, formulation 6 containing 3% *w*/*w* PVA 117, 0.5% *w*/*w* CMC, and 0.5% *w*/*w* HEC, and formulation 7 containing 3% *w*/*w* PVA 117, 1% *w*/*w* CMC, and 1% *w*/*w* HEC. All formulation contained 5% *w*/*w* PEG 400. ND was not determined. N/A data was unavailable due to the film unexpectedly pulling apart during the experiment. Different letters, a, b, c, d, e, and f denote significant differences among each sample.

**Table 4:** Characteristics of film-forming gel containing honey aromatic water with and without royal jelly.

Parameters	Film-Forming Gel Containing Honey Aromatic Water with and without Royal Jelly				
	6	6A	6R0.5	6R1	6R2
**Film-forming gel**					
Viscosity (mPa·s)	1.26 ± 0.01 ^a^	0.91 ± 0.02 ^c^	0.66 ± 0.03 ^e^	0.75 ± 0.05 ^d^	1.10 ± 0.03 ^b^
Spreadability (cm)	4.5 ± 0.1 ^c^	5.3 ± 0.0 ^b^	5.9 ± 0.3 ^a^	4.4 ± 0.2 ^c^	4.9 ± 0.1 ^b,c^
pH	5.0	4.5	4.5	4.5	4.5
Drying time (min)					
on glass slide	73.3 ± 1.2 ^a^	47.7 ± 0.6 ^c^	51.6 ± 0.6 ^b^	49.7 ± 1.6 ^b^	47.0 ± 1.0 ^c^
on piglet skin	19.4 ± 0.6 ^a^	17.4 ± 0.1 ^b^	14.7 ± 0.6 ^c^	10.4 ± 0.6 ^d^	18.4 ± 1.0 ^a,b^
**Film**					
Tensile strength (kg/cm^2^)	11.05 ± 2.54 ^a^	5.51 ± 0.58 ^c^	7.53 ± 0.81 ^b,c^	10.15 ± 0.76 ^a,b^	9.13 ± 0.07 ^a,b^
Elongation (%)	9.29 ± 1.01 ^a^	7.14 ± 0.00 ^b^	10.00 ± 4.04 ^a^	10.00 ± 0.00 ^a^	4.29 ± 0.00 ^c^
Young’s modulus (mPa)	1.18 ± 0.14 ^b^	0.77 ± 0.08 ^c^	0.80 ± 0.24 ^c^	1.02 ± 0.08 ^b,c^	2.13 ± 0.02 ^a^
Adhesion (N/mm)	19.8 ± 1.0 ^a^	2.1 ± 0.0 ^b^	1.6 ± 0.5 ^b^	2.5 ± 1.4 ^b^	19.8 ± 2.0 ^a^

NOTE: Formulation 6 containing 3% *w*/*w* PVA 117, 0.5% w/w CMC, and 0.5% *w*/*w* HEC, formulation 6A containing honey aromatic water; formulation 6R0.5 containing honey aromatic water and 0.5% *w*/*w* royal jelly; formulation 6R1 containing honey aromatic water and 1% *w*/*w* royal jelly; and formulation 6R2 containing honey aromatic water and 2% *w*/*w* royal jelly. All formulations contained 5% *w*/*w* PEG 400. Different letters, a, b, c, d, and e, denote significant differences among each sample.

**Table 5:** Ingredients of film-forming gel base formulations.

Ingredients	Amount (% *w*/*w*)						
	1	2	3	4	5	6	7
PVA 117	3	3	3	3	3	3	3
CMC	-	1	2	-	-	0.5	1
HEC	-	-	-	1	2	0.5	1
PEG 400	5	5	5	5	5	5	5
DI water	92	91	90	91	90	91	90

NOTE: PVA 117 = polyvinyl alcohol; CMC = carboxymethyl cellulose; HEC = hydroxyethyl cellulose; PEG 400 = polyethylene glycol 400; DI water = deionized water.

**Table 6:** Ingredients of film-forming gel containing honey aromatic water with and without royal jelly.

Ingredients	Amount (% *w*/*w*)				
	6	6A	6R0.5	6R1	6R2
Honey aromatic water	-	91	90.5	90	89
Royal jelly	-	-	0.5	1	2
PVA 117	3	3	3	3	3
CMC	0.5	0.5	0.5	0.5	0.5
HEC	0.5	0.5	0.5	0.5	0.5
PEG 400	5	5	5	5	5
DI water	91	-	-	-	-

NOTE: PVA 117 = polyvinyl alcohol; CMC = carboxymethyl cellulose; HEC = hydroxyethyl cellulose; PEG 400 = polyethylene glycol 400; DI water = deionized water.

## Data Availability

The data that support the findings of this study are available on request from the corresponding author.

## Supplementary Materials

The following supporting information can be downloaded at: https://www.mdpi.com/article/10.3390/gels9100816/s1, Figure S1: Non-sterile honey aromatic water (a), sterile honey aromatic water through filtration (b), and sterile honey aromatic water through autoclaving (c); Figure S2: The external appearance of non-sterile and sterile honey aromatic water by autoclaving and filtration kept at various temperatures observed at days 0, 1, 3, 7, 14, and 28; Figure S3: Spreadability measurement of film-forming gel using a TA.XT PLUS texture analyzer (Stable Micro Systems Ltd., Go-dalming, UK) equipped with a P/10 cylindrical Perspex (Lucite International Ltd., Queens Gate, UK) probe, which was lowered onto the surface of an upper glass plate with a constant speed of 1 mm/s; Figure S4: Peel test using a TA.XT PLUS texture analyzer (Stable Micro Systems Ltd., Godalming, UK) equipped with a heavy-duty platform (HDP/90) and A/TG tensile grips probe of a film from the film-forming gel base during the experiment (a and b) and after the film was unexpectedly pulled apart (c), as well as a film from the film-forming gel containing honey aromatic water and royal jelly before starting (d), during the experiment (e), and after the whole gel was completely peeled off (f).
