# Supplementary material for: Development of Film-Forming Gel Formulations Containing Royal Jelly and Honey Aromatic Water for Cosmetic Applications
Source: Gels. 2023 Oct 13;9(10):816. doi: 10.3390/gels9100816 (PMC10606181; doi:10.3390/gels9100816)
Supplement: Supplementary file 1 [file gels-09-00816-s001.zip › gels-2662997-supplementary.pdf]

gels

MDPI

Supplement material

Development of Film-Forming Gel Formulations Containing Royal Jelly and Honey Aromatic Water for Cosmetic Applications

Sirawut Thewanjutiwong [1], Patcharin Phokasem [2,3], Terd Disayathanoowat [3,4], Saranya Juntrapirom [5], Watchara Kanjanakawinkul [5] and Wantida Chaiyana [1,4,*]

1 Department of Pharmaceutical Sciences, Faculty of Pharmacy, Chiang Mai University, Chiang Mai 50200, Thailand; sirawut_t@cmu.ac.th
2 Office of Research Administration, Chiang Mai University, Chiang Mai 50200, Thailand; patcharin.ph@cmu.ac.th
3 Department of Biology, Faculty of Science, Chiang Mai University, Chiang Mai 50200, Thailand; terd.dis@cmu.ac.th
4 Research Center of Deep Technology in Beekeeping and Bee Products for Sustainable Development Goals: SMART BEE SDGs, Chiang Mai University, Chiang Mai 50200, Thailand
5 Chulabhorn Royal Pharmaceutical Manufacturing Facilities by Chulabhorn Royal Academy, Phlu Ta Luang, Sattahip, Chon Buri 20180, Thailand; saranya.jun@cra.ac.th (S.J.); watchara.kan@cra.ac.th (W.K.)
* Correspondence: wantida.chaiyana@cmu.ac.th; Tel.: +66-5394-4343

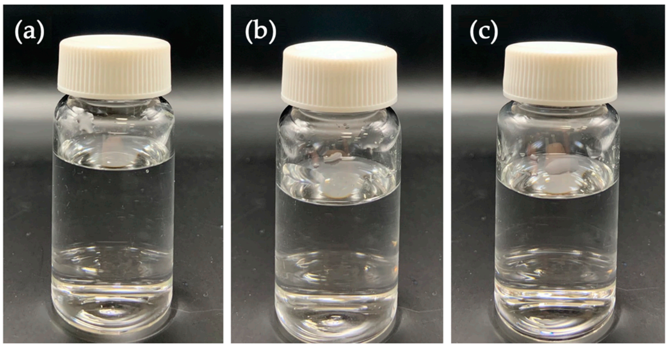

Figure S1. Non-sterile honey aromatic water (a), sterile honey aromatic water through filtration (b), and sterile honey aromatic water through autoclave (c).

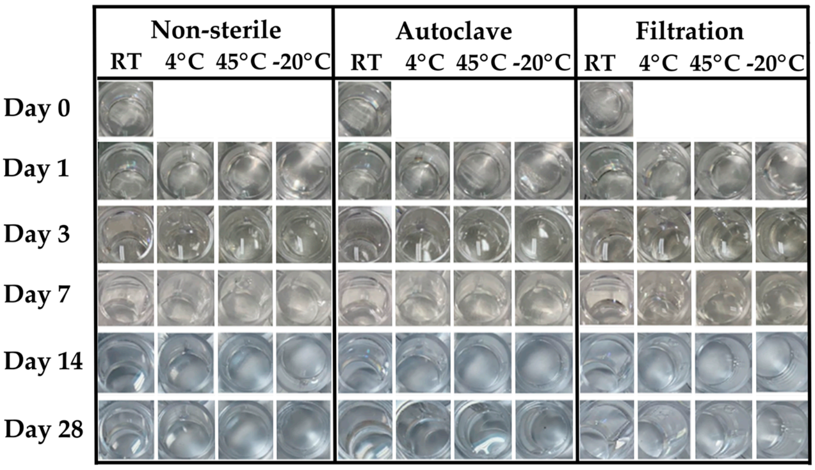

Figure S2. The external appearance of non-sterile and sterile honey aromatic water by autoclave and filtration kept at various temperatures observed at week 0, 1, 3, 7, 14, and 28.

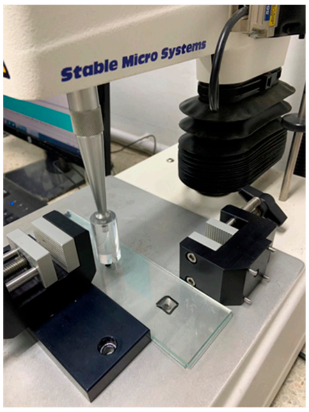

Figure S3. Spreadability measurement of film-forming gel using a TA.XT PLUS texture analyzer (Stable Micro Systems Ltd., Godalming, UK) equipped with a P/10 cylindrical Perspex (Lucite International Ltd., Queens Gate, UK) probe, which was lowered onto the surface of an upper glass plate with a constant speed of 1 mm/s.

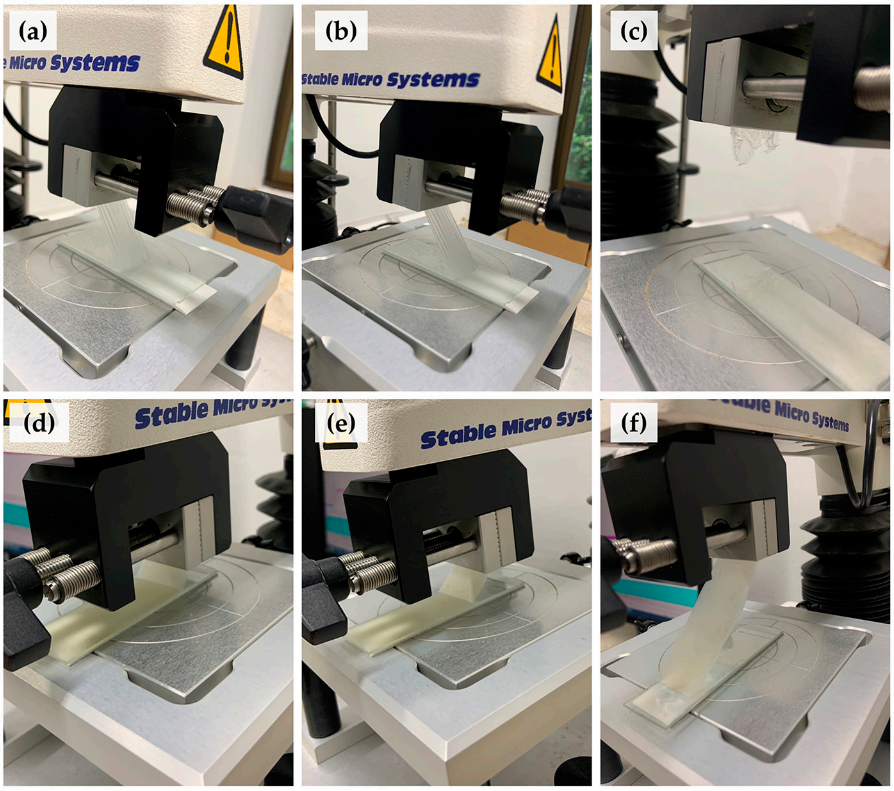

Figure S4. Peel test using a TA.XT PLUS texture analyzer (Stable Micro Systems Ltd., Godalming, UK) equipped with a 1 kg load cell, heavy-duty platform (HDP/90), and A/TG tensile grips probe of a film from the film-forming gel base during the experiment (a and b) and after the film was unexpectedly pulled apart (c), as well as a film from the film-forming gel containing honey aromatic water and royal jelly before starting (d), during the experiment (e), and after the whole gel was completely peeled off (f).
